# 

**Published:** 1912-10-05

**Authors:** 


### A NEW TRAVELLING SCHOLARSHIP.

MRS. RADCLIFFE CROCKER has given £1,500 to University College Hospital to endow a travelling scholarship in dermatology in memory of her husband, Dr. H. Radcliffe Crocker, M.D.Lond., F.R.C.P., formerly president of the Dermatological Society and for thirty years physician to the hospital. The scholarship, which carries with it a gold medal, will be awarded every five years.

### BUREAU OF INFORMATION.

### Rules for Correspondents.

1. Every letter, on whatever subject, **must be accompanied by the coupon** to be cut from the back cover (inside page) of The Hospital, current issue, and must contain the name and address of the correspondent with pseudonym for publication if desired.
2. Replies by post cannot be given, save under exceptional circumstances at the Editor's discretion.
3. Letters from Approved Homes in reply to special needs published in the Bureau should state terms and full particulars, and be sent prepaid under cover to the Editor of the Bureau with coupon and name enclosed for identification.
4. Proprietors of Homes which have not yet been entered on the List of Approved Homes, but have spare accommodation likely to suit special needs, are invited to write for an application form for registration. The fee for registration, which includes two announcements of the Home in the Bureau, is 5s.
5. Special needs of every description relating to those who are sick or in difficulties are made known in these columns **free of charge.**
6. All communications to be addressed to The Editor of The Hospital, 28 Southampton Street, Strand, London, W.C., and marked "Bureau of Information."

### Institutions with Pay Wards.

**Southport Homœopathic Cottage Hospital.**—Fleetwood Road. Medical, surgical, and occasionally epileptic cases. Terms: 31s. 6d. in large wards; from £3 3s. in private wards. Long cases received at a reduction. Sea air, very open situation.

### Needs and Helpers.

**Au Pair.**—Can any of our readers in the South or West of England offer a home for the winter to an experienced worker who is not strong, and wishes to give services in return for comfortable home? Age twenty-six. Active. Pleasant in appearance and manner. Not a trained nurse, but very useful and bright with convalescents or children. Please write L. A. G., c/o Bureau.

**Home for Defective Girl.**—It is very difficult to place a girl of twenty-one who is slightly deficient mentally and also slightly deformed, even though she is capable of responding to training. There are no institutions where such persons are received free. If she is fairly strong and can help in domestic work, some lady or home might be willing to receive her for two or three shillings a week in order to make her useful, under supervision, with house work. But such cases require constant watchfulness. We will forward any replies received.—Mrs. P., Eltham.

### Training and Employment.

**Dispensing in Canada and Australia**.—All the different provinces require persons dispensing drugs to be registered after examination; but for the most part they accept in lieu of examination the diploma of any other competent examining body as sufficient evidence of qualification. In some places the qualification of Minor or Major Diploma of the Pharmaceutical Society is mentioned as the alternative to examination. Write to the Society of Apothecaries, Water Lane, Blackfriars, and ask what Colonies accept their certificate. The *Canadian Nurse*, 408 McKinnon Building, Toronto, would be a good paper to advertise in. But we fear these institution posts are always filled up by persons from within or personally known to the medical staff, and we cannot recommend you to go out on the chance.—Cespicer.

**Canadian Nursing.**—Write to the Graduate Nurses' Home and Registry, 375 Langside Street, Winnipeg, and to the Central Registry of Nurses, 569 Bathurst Street,

Toronto, enclosing postal coupon for reply, and ask what prospect there would be for you of obtaining employment in those cities. We are making inquiries regarding another province in Canada for you, and will let you hear again.—B. J.

### Miscellaneous.

**Liquid Shaving Soap.**—The best thing for you to use would be ether soap. Presumably the reason why liquid soaps have been recommended by medical men is that by their use the danger of transferring skin disease from one client to another on the shaving-stick is avoided. This danger is certainly a real one; bacteriological researches have proved this. Moreover, ether is itself antiseptic to some extent, and it dissolves the grease off the surface of the skin, thus helping the cleansing action of the soap. You will find it dries rather quickly.—"Anxious."

**Book on Home Nursing.**—We can recommend "Some Practical Instructions on Home Nursing for Untrained Folk," by Miss Esther Young. (The Scientific Press. Price 6d.) The catalogue is being sent on.—Nurse J. G.

### Approved Homes Added to the List, April to September, 1912.

Note.—*No Home is added to the List without direct medical and other testimony to its good management.*

**Blackpool and St. Anne's.**—Nurses' and Nursing Homes: 112 Hornby Road, Blackpool; 17 Fairhaven Road, St. Anne's-on-the-Sea. Superintendents: Miss Mary M. Strange and Sister Grace Strange. Medical, surgical, slight mental, maternity, chronic, tuberculosis, and convalescent cases received. Near sea. Garden and greenhouse.

**Brighton.**—3 Sudeley Street, Brighton. Proprietress: Nurse Dunkley, trained maternity nurse. Maternity, medical, and convalescent cases. An excellent home for patients of moderate means; highly recommended for scrupulous cleanliness and kind attention.

**Chesterfield.**—120 Old Hall Road, Chesterfield. Proprietress: Sister "Pam," trained nurse. Good house, standing in own grounds, free from noise. Medical, surgical, and nerve cases received. Terms very moderate.

**Essex.**—Co-operative Sanatoria, New Lodge and Leon House, Billericay. Matron: Mrs. Edwards, cert. M.P.A. For the reception at very moderate charges of male patients suffering from slight mental ailments or epilepsy. Conducted on co-operative principles, the interest on share capital being limited to 5 per cent. Under medical direction. Fully qualified staff of nurses and attendants. Large grounds, with farm and recreation fields. Terms from 16s. to 42s. weekly, including laundry and all expenses.

**Hampshire.**—Private Home, Ashcombe House, New Milton, Hants. Mrs. Brockell receives chronic, convalescent, and nerve cases. Invigorating air and mild climate in winter.

**Herne Bay.**—Sea View House, Canterbury Road, Herne Bay. Proprietress: Mrs. Ingle. Bright and airy premises facing the sea on the East Cliff. It is well adapted for nerve patients, with whom Mrs. Ingle has had wide experience. Medical and surgical patients received also. Massage, rest-cures.

**Ireland.**—1 Esplanade Terrace, Bray, Co. Wicklow. Principal: Nurse Roberts-Murray, cert. Rotunda Hospital, Dublin. Obstretric, non-infectious, medical, slight mental, or nerve cases. Sea and mountain air. Established six years. Moderate terms.

**Kent.**—Mrs. Newman, Normanton, Chelsfield, Kent, can receive lady or gentleman, at moderate terms, requiring quiet home and freedom from worry. Very suitable for after-care. Pretty country and good air.

**London.**—Lyndhurst Lodge, Whitehorse Lane, South Norwood, London, S.E.—Proprietors: Mr. and Mrs. Aldridge, trained mental nurses. For slight mental, feeble, paralytic, epileptic, or nerve cases. Two acres of wooded ground, three hundred and fifty feet above sea-level. Croquet, bowls, etc. Private rooms. Terms very moderate.

**London.**—5 Highbury Quadrant, London, N. Proprietresses: Miss Evelyne Johns, Miss Florence Johns. Certificated, trained nurses. Medical, surgical, maternity, slightly mental, nerve cases. Well situated on high ground in quiet situation. Garden. Patients visited by their own doctors.

**London.**—28 Cleveland Gardens, Hyde Park, W. Proprietress: Miss L. M. Patten. Maternity, heart, rest-cure, chronic cases. All branches of massage; Swedish exercises; Nauheim treatment. Close to Kensington Gardens. Family life, if desired. Specially adapted for children.

**London.**—High-class Nursing Home, 1 Westbourne Square, W. Proprietress: Miss Mahomy. Medical, surgical, maternity cases. Long established, and thoroughly well equipped.

**London.**—23 Westbourne Gardens, Bayswater, London, W. Proprietresses: Miss Watson and Miss Mudd. Excellent accommodation for medical, surgical, maternity, rest-cure, and chronic patients. Personal supervision. Special attention to good dieting. Quiet locality.

**London.**—3 Lorn Road, Brixton, S.W. Nurse McCoy receives slight mental and nerve cases. Massage, electrical and Nauheim treatment. Experienced and successful with nervous patients. Nice garden and every comfort.

**London.**—Fulham Hydropathic Institute, 14, 16 Whittingstall Road, Munster Park, Fulham, S.W.—Principal: Miss Hannaford, trained mental nurse, assisted by masseur for male patients and by masseuse with certificates in massage. The Nauheim treatment and medical electricity. Patients received under medical direction for treatment by the Weir-Mitchell and also by the Mustard Pack treatment. Slight mental and medical cases can also be received at very moderate terms. Inspected and approved by Medical Officer of Health for district.

**Lowestoft.**—The Alexandra Home, 407-409 London Road, South Lowestoft. Proprietresses: Nurses E. and M. Manwaring. Medical, surgical, maternity, chronic, and convalescent cases. Bracing air, near sea. Good operating theatre. Patients visited by their own doctors.

**Middlesex.**—Greylands, Hatchend. Patients received for change and rest or open-air cure. Nice garden; good balconies for out-door sleep, if desired. Quiet situation. Terms very moderate.

**Peterborough.**—Rothesay Villas, 73 Lincoln Road. Proprietress: Miss Warburton. Medical, surgical, maternity cases. Special experience with Weir-Mitchell treatment. Chronic patient can be taken at low terms. Fully-trained nurses supplied from 31s. 6d. weekly.

**St. Leonards-on-Sea.**—87 Marina, St. Leonards-on-Sea. Superintendent: Miss Greenwood. Medical, surgical, slight mental, chronic, and convalescent cases. Very kind care taken of the aged and helpless.

### Appointments.

Dr. M. Cohen, resident medical officer at the Hull Workhouse, has been appointed senior tuberculosis officer to Hull.

Dr. Davis and Dr. Campbell have been elected house surgeons to the Royal Portsmouth Hospital for the ensuing six months.

Mr. W. P. Hogg, M.B., Ch.B., Aberdeen, has been appointed house physician to the Leicester Royal Infirmary. Mr. Hogg vacates the post of assistant house physician.

The Council of the University of Sheffield has made the following appointments : (1) Mr. Henry Roy Dean, M.A., M.D. (Oxon.), M.R.C.P. (London), to the Joseph Hunter Chair of Pathology in place of Professor J. M. Beattie, who has been appointed to the Chair of Bacteriology in the University of Liverpool. Dr. Dean is at present assistant bacteriologist to the Lister Institute of Preventive Medicine. (2) Mr. Leonard Southerns, B.A. (Cantab.), B.Sc. (London), to the post of junior lecturer and demonstrator in physics, vice Dr. J. Robinson, resigned. Mr. Southerns is at present chief assistant at the Eskdalemuir Observatory, N.B. The Council received the resignation of Professor F. W. Hardwick of the professorship of mining owing to his retirement from active work. Professor Hardwick has been on the staff since October 1891. Dr. Arthur Hall has been appointed Dean of the Faculty of Medicine in place of Professor Beattie, resigned.

### Gifts and Bequests.

The City Corporation has contributed £105 to the Hospital Sunday Fund.

The Clapton Orient Football Club have sent £20 to the City of London Chest Hospital, Victoria Park.

Mr. William Yates, of Shepperton-on-Thames, retired mechanical engineer, has bequeathed £2,000 to the Blackburn Royal Infirmary.

The Rev. Arthur White, of Exmouth, late Fellow of Magdalene College, Cambridge, has bequeathed £100 each to the Exmouth District Nurses' Fund, the Exmouth Cottage Hospital, and the Exmouth Dispensary.

The late Mr. James Slocombe, of Cardiff, has bequeathed 1,000 guineas to King Edward VII.'s Hospital, Cardiff, to endow a bed, to be named after the testator and his wife, in such a ward as the same should be most needed. Mr. Slocombe, who was eighty years of age at his death, had been a consistent annual subscriber to the hospital for over forty years.

